# Prognostic role of neutrophil lymphocyte ratio and mean platelet volume in Bell’s palsy: Comparison of diabetic and non-diabetic patients

**DOI:** 10.1016/j.bjorl.2021.10.003

**Published:** 2021-11-15

**Authors:** Serhat İnan, Sabuhi Jafarov

**Affiliations:** Baskent University, Faculty of Medicine, Department of Otolaryngology, Head and Neck Surgery, Ankara, Turkey

**Keywords:** Bell’s palsy, Neutrophil lymphocyte ratio, Diabetes mellitus, Mean platelet volume, Facial paralysis

## Abstract

**Objectives:**

A low Neutrophil Lymphocyte Ratio (NLR) has been shown to be associated with good prognosis in Bell’s Palsy (BP). However, the effect of chronic diseases that may affect the NLR, including Diabetes Mellitus (DM), has not been clarified in this context. This study aimed to evaluate the relationship between NLR and Mean Platelet Volume (MPV) in BP according to whether it is accompanied by DM, and their relationship with prognosis.

**Methods:**

A prospective observational study was conducted from May 2014 to May 2020 in a tertiary referral center, of all 79 consecutive participants diagnosed with BP in department of otolaryngology and 110 consecutive healthy participants admitted to the check-up unit. Patients diagnosed with BP were divided into two groups according to whether they were diagnosed with DM: diabetic BP patients (DM-BP, n = 33) and non-diabetic BP patients without any chronic disease (nonDM-BP, n = 46). Neutrophil (NEUT) and Lymphocyte (LYM) counts, and Mean Platelet Volume (MPV) were assessed from peripheral blood samples, and the NLR was calculated. Prognosis was evaluated using the House–Brackmann Score (HBS) six months after diagnosis.

**Results:**

The mean NLR was 2.85 ± 1.85 in BP patients and 1.69 ± 0.65 in the control group. The mean NLR was significantly higher in BP patients than healthy controls (*p* < 0.001). The mean NLR was 2.58 ± 1.83 in the nonDM-BP group, 3.23 ± 1.83 in the DM-BP group, and 1.69 ± 0.65 in the control group. The NLR was significantly higher in the nonDM-BP and DM-BP groups than in the control group (*p* < 0.05). The recovery was 90% according to the HBS. The optimal cut-off value was 2.41 (*p* = 0.5).

**Conclusion:**

The NLR was increased in both diabetic and non-diabetic BP and had similar prognostic value in predicting the HBS before treatment in diabetic and non-diabetic patients with BP. MPV wasn’t significantly different in diabetic and non-diabetic BP patients compared with the normal population.

## Highlights

• NLR is a valuable prognostic indicator for diabetic and non-diabetic patients with BP.
• NLR is significantly higher in the nonDM-BP and DM-BP groups than in healthy controls.
• A high NLR is associated with higher stage HBS before treatment.
• MPV is not significantly different in diabetic and non-diabetic BP patients.
• The recovery rate according to the HBS was 90% in BP, and not affected by diabetes.

## Introduction

Bell’s Palsy (BP) is an idiopathic peripheral Facial Paralysis (FP) characterized by acute unilateral weakness of the facial muscles. BP is the most common cause of FP. The annual incidence is between 15 and 30 per 100,000 people.^1^ Herpes simplex, ischemia, inflammation, and immune disorders have been suggested as involved in BP pathogenesis.^2^ It has been frequently suggested that FP may arise as a result of demyelination secondary to acute inflammation. Histological evidence supporting this view consists in the expansion of the internal area with the accumulation of inflammatory cells containing macrophages between the internal acoustic canal and the stylomastoid foramen in the facial nerve.^3^ The facial nerve has a rigid epineurium and abundant vascular resources. However, vasospasms may cause decreased blood flow and ischemic neuropathy in the facial nerve after transient ischemia and reperfusion in some clinical conditions, such as diabetes mellitus. Macrophage activation and infiltration and acute inflammation are triggered in this case.^4^ The frequency of diabetes mellitus varies greatly among patients with BP, and there is no consensus on whether Diabetes Mellitus (DM) is a risk factor or a predisposing factor for BP, although many studies have discussed this issue.^5^, ^6^ Systemic steroids with or without additional antiviral agents have been recommended as standard therapy for BP in previous randomized controlled trials. Approximately 70% of patients with BP show complete recovery without receiving any treatment^7^; this rate increases to 90% with treatment.^8^

The House–Brackmann Score (HBS) is used to evaluate paralysis and is associated with prognosis.^9^ Electroneurography (ENoG) is often used to predict prognosis and make additional decisions, including the use of facial nerve decompression surgery. However, the applicability of ENoG and Electromyography (EMG), the primary methods used in clinical practice, is relatively difficult. Some of the positive prognostic factors that may be associated with improvement in facial function are young age, lower HBS, good electromyography results, absence of diabetes, and control of hypertension.^10^

A high Neutrophil-Lymphocyte Ratio (NLR) is considered a reliable etiological indicator of disease severity in inflammatory disorders.^11^, ^12^ Studies have shown that the mean NLR and neutrophil values were significantly higher in adult and pediatric patients with BP than in healthy controls.^13^, ^14^, ^15^, ^16^ However, the effect of chronic diseases that may affect the NLR value has not been clarified in studies showing the association between low NLR and good prognosis in BP.

DM is one of the diseases that have been implicated in the etiology and prognosis of BP. NLR has also been shown to be increased in patients with diabetes. Likewise, high Mean Platelet Volume (MPV) values demonstrate increased platelet activity in patients with diabetes and are associated with insulin resistance. Based on these findings, this study aimed to evaluate the NLR and MPV in BP according to whether it is accompanied by DM, and their relationship with prognosis.

## Methods

This study was approved by the Başkent University Institutional Review Board and Ethics Committee (Project nº KA 15/245) and was conducted in accordance with the Declaration of Helsinki and its later amendments. Written informed consent was obtained from all participants. The study prospectively included 79 consecutive participants diagnosed with BP between May 2014 and May 2020 and 110 consecutive healthy participants admitted to the check-up unit. Patients diagnosed with BP were divided into two groups depending on the concomitant presence of DM: diabetic BP patients (DM-BP) and non-diabetic BP patients (nonDM-BP). All patients with FP were evaluated by an otorhinolaryngologist. FP grade was determined using the HBS by the same two researchers. All patients were evaluated by MRI using internal acoustic canal gadolinium contrast. Neutrophil (NEUT) and Lymphocyte (LYM) counts, and Mean Platelet Volume (MPV) were assessed using an Abbott CELL-DYN Ruby System (Abbott Diagnostics, Santa Clara, CA, USA) from peripheral blood samples obtained from patients with FP developed within 3 days or less. The NLR, a marker of subclinical inflammation, was calculated by dividing the number of neutrophils by the number of lymphocytes. The percentage of glycated Hemoglobin (HbA1c) of patients in the DM-BP group was measured. All diabetic patients required subcutaneous insulin injections during the treatment for blood glucose control. Routine prednisolone treatment was administered to all patients (prednisolone 250 mg IV on day 1, 150 mg IV on day 2, 80 mg PO 2 days, 64 mg PO 2 days, 48 mg PO 2 days, 32 mg 2 days, 16 mg PO 2 days). The prognosis was evaluated using the HBS six months after diagnosis. According to the final evaluation of facial functions, H-B grades I and II were considered as a recovery while HB grade over II considered as non-recovery.

The exclusion criteria were as follows: polyneuropathies such as central induced paralysis, cerebellopontine corner tumor, herpes zoster oticus, otitis media, Guillain–Barre syndrome, recurrent BP, bilateral FP, traumatic FP, iatrogenic FP; more than 3 days from the onset of FP, follow-up period of less than 6 months, chronic renal failure, chronic liver failure, previous transplant, chronic arterial disease, inflammatory bowel disease, vasculitis, malignancies, asthma patients, pregnant women; botulinum toxin administration, and use of steroids, antihistamines, chemotherapy, antibiotics, or anti-inflammatory drugs; and grade II HBS at the time of admission.

### Statistical analysis

SPSS 23.0 package program (Statistical Package for the Social Sciences) was used for statistical analysis of the data. Categorical measurements were presented as number and percentage while continuous measurements were expressed as mean and standard deviation (median and minimum-maximum, where necessary). Chi-Square test and Fischer’s precision test were used to compare categorical variables. Shapiro–Wilk test was used to determine whether the parameters in the study showed normal distribution. In comparing the continuous measurements between the groups, the distributions were checked, and independent Student *t*-test was used for the parameters with normal distribution, Mann Whitney *U* test was used for the parameters not showing normal distribution, and Kruskall Wallis test was used for more than two variables. The relationship between quantitative variables was examined with Pearson and Spearmean correlation analyses. Analysis of variance (ANOVA) was used to check if the means of two or more groups are significantly different from each other. The optimal cut-off value was determined using Receiver Operating Characteristic (ROC) curve. The level of statistical significance was accepted as 0.05 in all tests.

## Results

A total of 189 patients, including 46 (26 men) in the nonDM-BP group; 33 (15 men) in the DM-BP group; and 110 (63 men) in the healthy control group participated in the study. There were no differences among the groups in terms of sex distribution. The mean age was 41 ± 15 years in the non-DM-BP group, 58 ± 15 years in the DM-BP group, and 43 ± 12 years in the control group. The mean age was significantly higher in the DM-BP and nonDM-BP groups than in the control group (*p* < 0.001), (Table 1). There was no difference between the nonDM-BP and DM-BP groups in terms of FP side (*p* = 0.454). The mean HbA1c in the DM-BP group was 6.90 ± 0.48% (range 5.78–8.10).

**Table 1:** Demographic characteristics and peripheral complete blood count values.

Group	N (male/female)	Age	LEUK	NEUT	LYMP	NLR	MPV
nonDM-BP	46 (26/20)	41 ± 15	8.36 ± 2.22	5.22 ± 1.86	2.37 ± 0.91	2.58 ± 1.83	8.67 ± 1.26
DM-BP	33 (15/18)	58 ± 15^a^	8.57 ± 2.32	5.80 ± 2.07	2.11 ± 0.90	3.23 ± 1.83	8.36 ± 1.33
Control	110 (63/47)	43 ± 12	6.83 ± 1.5^a^	3.73 ± 1.07^a^	2.31 ± 0.66	1.69 ± 0.65^a^	8.54 ± 1.78
Total	189	45 ± 14	7.51 ± 2.04	4.45 ± 1.73	2.29 ± 0.77	2.18 ± 1.41	8.54 ± 1.59

nonDM-BP, Non-diabetic Bell’s Palsy patients; DM-BP, Diabetic Bell’s Palsy patients; LEUK, Leukocyte Count; NEUT, Neutrophil count; LYMP, Lymphocyte count; NLR, Neutrophil Lymphocyte Ratio; MPV, Mean Platelet Volume.

a *p* < 0.005.

**Figure 1:**
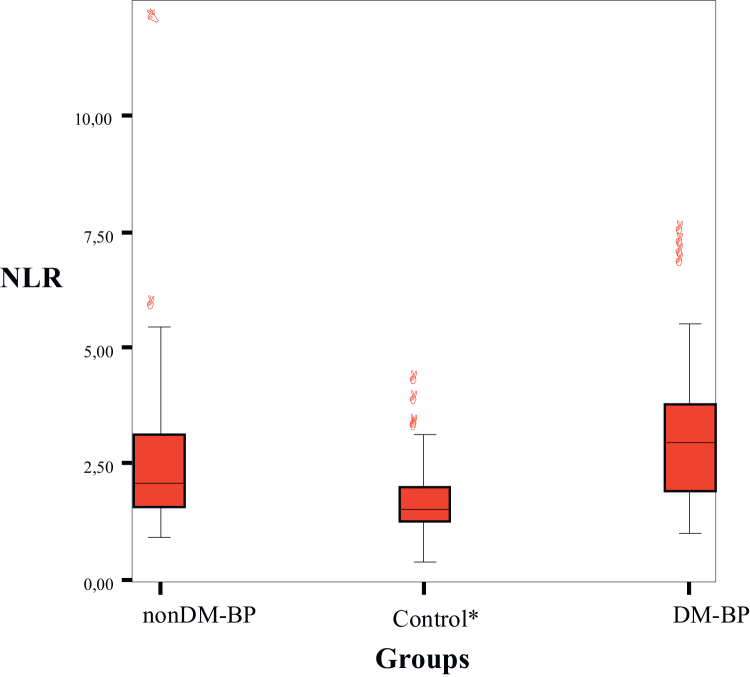
NLR comparison in diabetic and non-diabetic Bell’s palsy patients. NLR, Neutrophil Lymphocyte Ratio; nonDM-BP, Non-Diabetic Bell’s Palsy Patients; DM-BP, Diabetic Bell’s Palsy Patients; *p < 0.005.

The mean NLR was 2.85 ± 1.85 in BP patients and 1.69 ± 0.65 in the control group. The mean NLR was significantly higher in BP patients than healthy controls (*p* < 0.001). The mean NLR was 2.58±1.83 in the nonDM-BP group, 3.23 ± 1.83 in the DM-BP group, and 1.69 ± 0.65 in the control group. The NLR was significantly higher in the nonDM-BP and DM-BP groups than in the control group (*p* < 0.05). The mean NLR was not significantly different between the DM-BP and nonDM-BP groups (*p* = 0.073), (Fig. 1). The leukocyte count was significantly higher in the DM-BP and nonDM-BP groups than in the control group (*p* < 0.001). MPV and NEUT and LYM counts were not different among the groups (*p* > 0.05). NLR and HbA1c were positively correlated in the DM-BP group (*r* = 0.758, *p* < 0.001).

**Figure 2:**
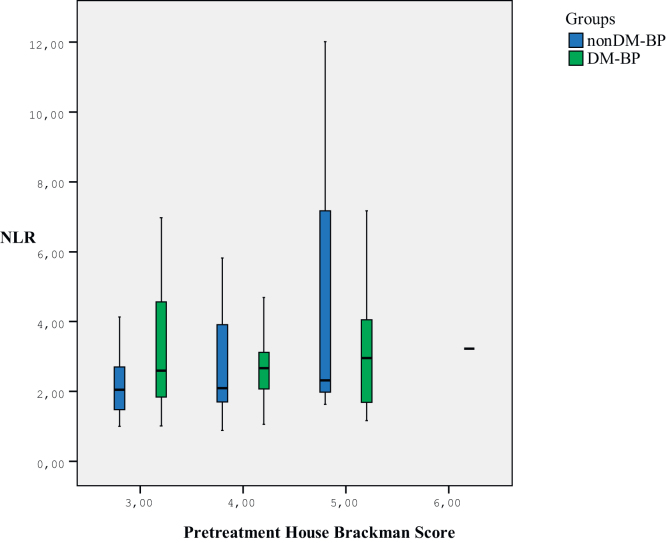
NLR comparison according to House–Brackman score before treatment in Bell’s palsy patients with and without diabetes. NLR, Neutrophil Lymphocyte Ratio; nonDM-BP, Non-Diabetic Bell’s Palsy Patients; DM-BP, Diabetic Bell’s Palsy Patients.

**Figure 3:**
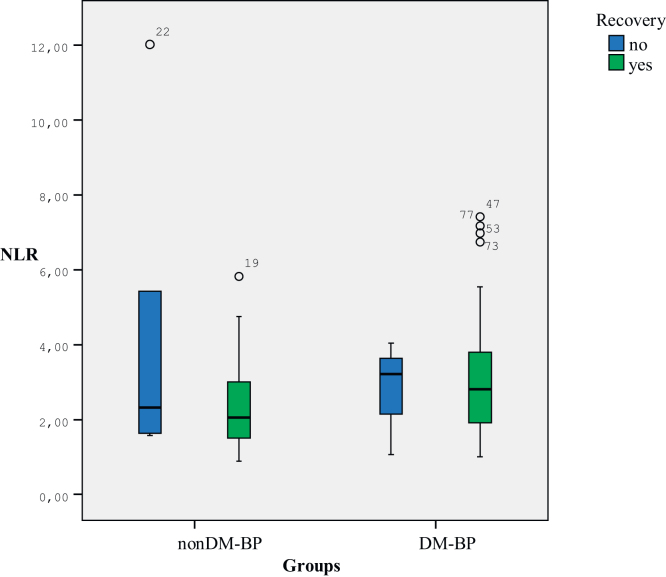
Comparison of NLR in diabetic and non-diabetic Bell’s palsy patients according to their recovery status. NLR, Neutrophil Lymphocyte Ratio; nonDM-BP, Non-Diabetic Bell’s Palsy Patients; DM-BP, Diabetic Bell’s Palsy Patients.

The HB grade was classified as grade III in 28 patients, grade IV in 15 patients, and grade V in 3 patients in the DM-BP group. Five patients were classified as grade III, 11 patients as grade IV, 6 patients as grade V, and 1 patient as grade VI in the DM-BP group. There was no difference in either pre-treatment or post-treatment HB grades between the nonDM-BP and DM-BP groups (*p* > 0.05), (Fig. 2). The recovery rate according to the HBS was 90%, with no difference between the DM-BP and nonDM-BP groups (*p* = 1). The mean NLR was 2.73 ± 1.54 in BP patients with recovery and 3.91±3.58 in BP patients with non-recovery. The mean NLR was significantly higher in BP with non-recovery group than BP with recovery (*p* = 0.006). ROC curve analysis of NLR to predict recovery in BP showed the optimal cut-off as 2.41 (AUC 0.573 [0.331–0.815]), sensitivity 50% and specificity 54.9%), (*p* = 0.5). The NLR increased as the HBS increased before treatment in BP patients (*r* = 0.218, *p* = 0.054), (Fig. 3). The prognostic power of the NLR is unchanged between diabetic and non-diabetic BP patients (*p* = 0.089).

## Discussion

Özler et al. reported that the NLR was higher in HB grade V patients before treatment compared with patients with HB grade III, IV, and VI.^17^ Kılıçkaya et al.^18^ and Kum et al.^14^ showed a positive correlation between HBS and NLR: The NLR increased with the HB stage before treatment, similar to the results of our study. Atan et al.^13^ reported no statistically significant relationship between the NLR and pre-treatment HB score in adult patients and Eryılmaz et al.^15^ reported similar results in pediatric patients. The NLR was found to be significantly higher in nonDM-BP and DM-BP patients than in the control group in the present study. The NLR tended to be higher in the DM-BP group than in the nonDM-BP group although this increase was not statistically significant. These results show a change in a peripheral blood signature of inflammatory processes in the pathology of facial paralysis.

The variability of inclusion and exclusion criteria in studies investigating the role of NLR in BP affects the reliability of the results.^19^ Some of these studies included patients with DM.^18^, ^20^ whereas this criterion was not specified in some studies.^14^, ^16^ However, some studies that found NLR to be higher in patients with BP than in the normal population did not specify whether other diseases affecting the NLR, such as liver, kidney, and heart disease were excluded.^20^ In our study, we excluded all possible inflammatory diseases other than DM to reveal the effect of DM on NLR in patients with BP.

Various factors affecting BP prognosis have been suggested, including age, DM, hypertension, and hypercholesterolemia. Takemoto et al. reported a relationship between poor recovery and DM.^21^ However, other studies reported no differences in the recovery rates of patients with and without DM. Among these, Sittel reported a recovery rate of 87.5% in 16 diabetic BP patients treated with an initial steroid dose of 250 mg.^22^ Kanazawa et al.^23^ found no significant differences between pre-treatment and 1-month post-treatment HB stages in BP patients with and without DM. Similarly, Eliçora et al.^24^ reported that DM had no effect on BP recovery time. The improvement in diabetic BP patients was found to be similar to that in non-diabetic BP patients, similar to the findings of the present study.

The NLR has been reported to be higher in patients with type 2 DM than in those without diabetes, and this increase has been reported to be associated with high insulin resistance.^25^, ^26^ Sefil et al.^27^ reported that an increased NLR was associated with HbA1c values above 7%. Increased NLR was reported to be a reliable marker of early-stage diabetic neuropathy in another study, and the same study found increased albuminuria in diabetic neuropathy with increased NLR.^28^ NLR has been recently reported to be associated with arterial stiffness, and therefore an increase in type 2 DM and diabetic retinopathy.^29^ HBA1c and NLR were positively correlated in our study.

The MPV parameter, indicating platelet volume, can also be associated with microvascular thrombotic disease. The correlation between MPV and stroke has been demonstrated in the literature.^30^ MPV is associated with microvascular obstruction. Some studies have suggested that high MPV values demonstrate increased platelet activity in patients with diabetes and are associated with insulin resistance.^31^ The correlation between MPV levels and Bell’s palsy has not been documented in the literature. There were no significant intergroup differences in MPV values in the present study, in agreement with the results reported in the literature.

## Conclusion

NLR has a similar prognostic significance for diabetic and non-diabetic patients with BP, and a high NLR is associated with higher stage HBS before treatment. MPV was not significantly different in diabetic and non-diabetic BP patients compared with the normal population.

## Conflicts of interest

The authors declare no conflicts of interest.
